# Cupredoxin engineered upconversion nanoparticles for ratiometric luminescence sensing of Cu^2+^†

**DOI:** 10.1039/c9na00168a

**Published:** 2019-05-13

**Authors:** Chang Liu, Yingjie Yu, Daquan Chen, Jian Zhao, Yang Yu, Lele Li, Yi Lu

**Affiliations:** School of Pharmacy, Collaborative Innovation Center of Advanced Drug Delivery System and Biotech Drugs in Universities of Shandong, Yantai University Yantai 264005 China; CAS Key Laboratory for Biomedical Effects of Nanomaterials and Nanosafety, CAS Center for Excellence in Nanoscience, National Center for Nanoscience and Technology Beijing 100190 China lilele@nanoctr.cn; Department of Biomedical Engineering, Tufts University Medford MA 02155 USA; University of Chinese Academy of Sciences Beijing 100049 China; Department of Chemistry, University of Illinois at Urbana–Champaign Urbana Illinois 61801 USA yi-lu@illinois.edu

## Abstract

The NIR excitation and large anti-Stokes shift of upconversion nanoparticles (UCNPs) have made them an ideal choice as biological nanoprobes. A key challenge in the field is to confer biorecognition units to UCNPs so that they can be used to probe specific targets in biological systems. While various agents have been combined with UCNPs to meet such a challenge, most studies are limited to small molecules, while biomolecules such as metalloproteins that possess much higher affinity and selectivity for metal ions have not been explored. Herein we demonstrate that fusion of zwitterion-coated UCNPs with azurin, a member of a family of redox-active copper proteins called cupredoxins that play important roles in diverse biological functions, can serve as an ideal platform for the label-free upconversion luminescence sensing of Cu^2+^ with a ratiometric response. The selective binding of apo-azurin with Cu^2+^ induces a significant absorbance at about 625 nm, and hence decreases the red emission of the UCNPs. In contrast, the green emission of the UCNPs remains constant and acts as an internal standard reference for the ratiometric sensing of Cu^2+^. This approach opens a new window for the development of assays for biosensing based on a combination of specific metalloproteins with UCNPs.

## Abstract 2

The integration of zwitterion-coated UCNPs with azurin, a member of a family of redox-active copper proteins, can serve as an ideal platform for the label-free upconversion luminescence sensing of Cu^2+^ with a ratiometric response.

## Graphical abstract

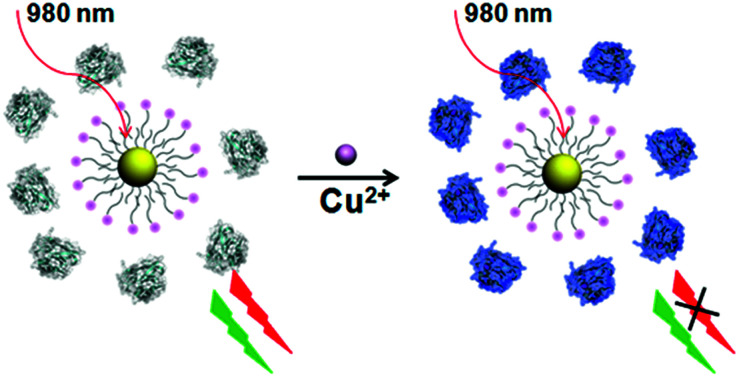

## Introduction

**Fig. 1:**
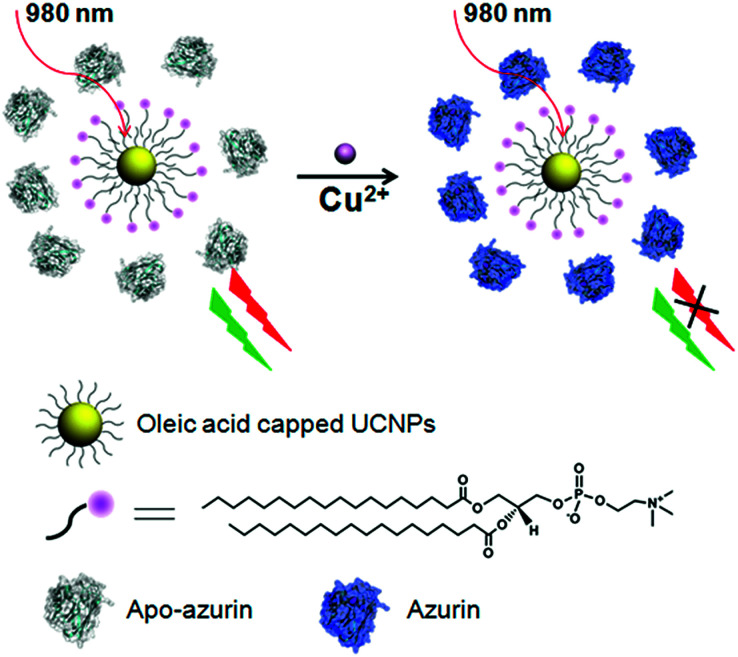
Schematic illustration of the ratiometric upconversion luminescence sensing of Cu^2+^ based on an azurin–UCNP biosensor.

Lanthanide ion doped upconversion nanoparticles (UCNPs) are a new class of luminescent probes that convert NIR excitation light into shorter wavelength luminescence *via* multiphoton absorption and energy transfer processes, which can achieve higher-contrast optical sensing and imaging as well as deeper tissue penetration due to a suppression of autofluorescence from biomolecules and a light scattering background.^1–4^ In contrast to organic fluorophores and quantum dots (QDs), UCNPs exhibit neither photoblinking nor photobleaching, and their rare earth components are much less toxic than the heavy metals within QDs.^1*a*,*b*^ Due to these advantages, UCNPs have attracted attention from researchers in fields such as sensing,^2*d*–*g*,5–7^ imaging,^1*b*,3*a*–*d*^ and therapeutics.^3*a*,4*b*^ Within this context, a number of UCNP-based sensor systems have been reported to detect various targets, such as metal ions,^5^ anions,^6^ and biomolecules.^7^ The key for the construction of such sensors is the integration of UCNPs with suitable molecules that possess specific recognition sites. However, the molecules used are mainly limited to small organic molecules. Since nature has evolved a number of tight regulatory proteins with high selectivity and sensitivity for binding of targets such as metal ions, the usage of these proteins as a sensor tool is currently attracting much interest.^8^ A major limitation of this design is the transformation of the binding events into an easily detectable signal. In this report, UCNPs were coupled with a unique redox protein (azurin) as a ratiometric sensor for Cu^2+^ sensing (Fig. 1).

As a redox-active metal ion, copper is a cofactor involved in many important biological processes, such as oxidative scavenging and respiration.^9^ However, at elevated concentrations, it can cause a number of severe health problems such as Menkes and Wilson's diseases, Alzheimer's disease, and liver or kidney damage.^9*c*^ Therefore, the design of fluorescent copper sensors has become a hot topic in analytical chemistry and many effective metal ion sensors based on small organic molecules, proteins, and DNA have been reported.^8,10^ Despite the progress, facile on-site and real-time detection and quantification of Cu^2+^ in aqueous solution remain complicated due to its paramagnetic quenching effects on fluorophores.^11^ Alternatively, ratiometric sensors with a change in the ratio of multiple emission bands provide built-in correction for environmental effects, and thus are free of such quenching problems.

## Results and discussion

**Fig. 2:**
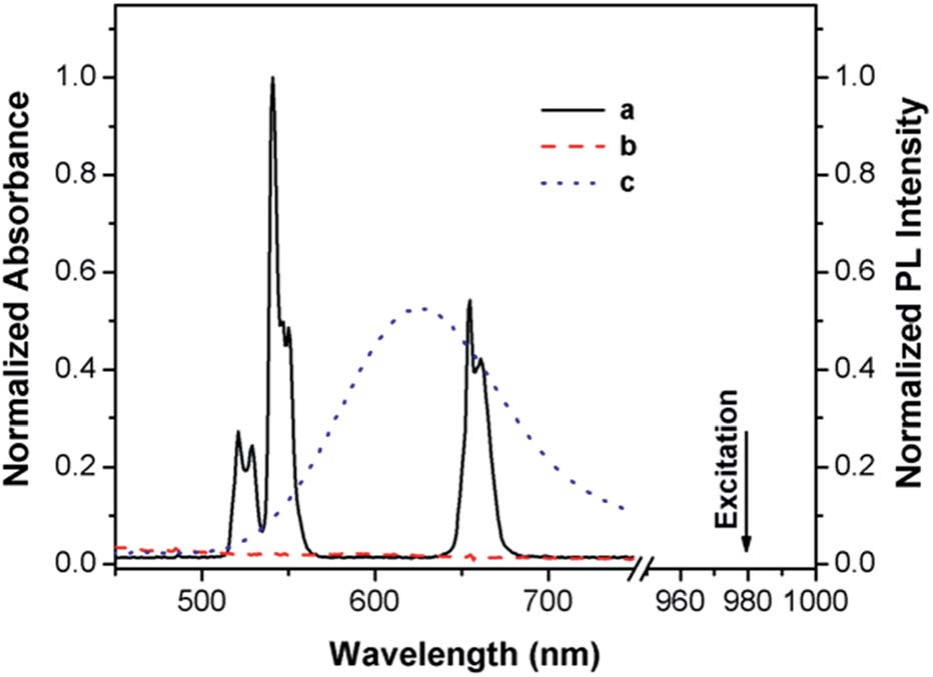
UCL spectrum of DSPC-coated NaYF_4_:20%Yb,2%Er (DSPC–UCNPs) under excitation from a 980 nm laser (a), and UV-vis spectra of the protein apo-azurin in the absence (b) and presence of Cu^2+^ (c).

**Fig. 3:**
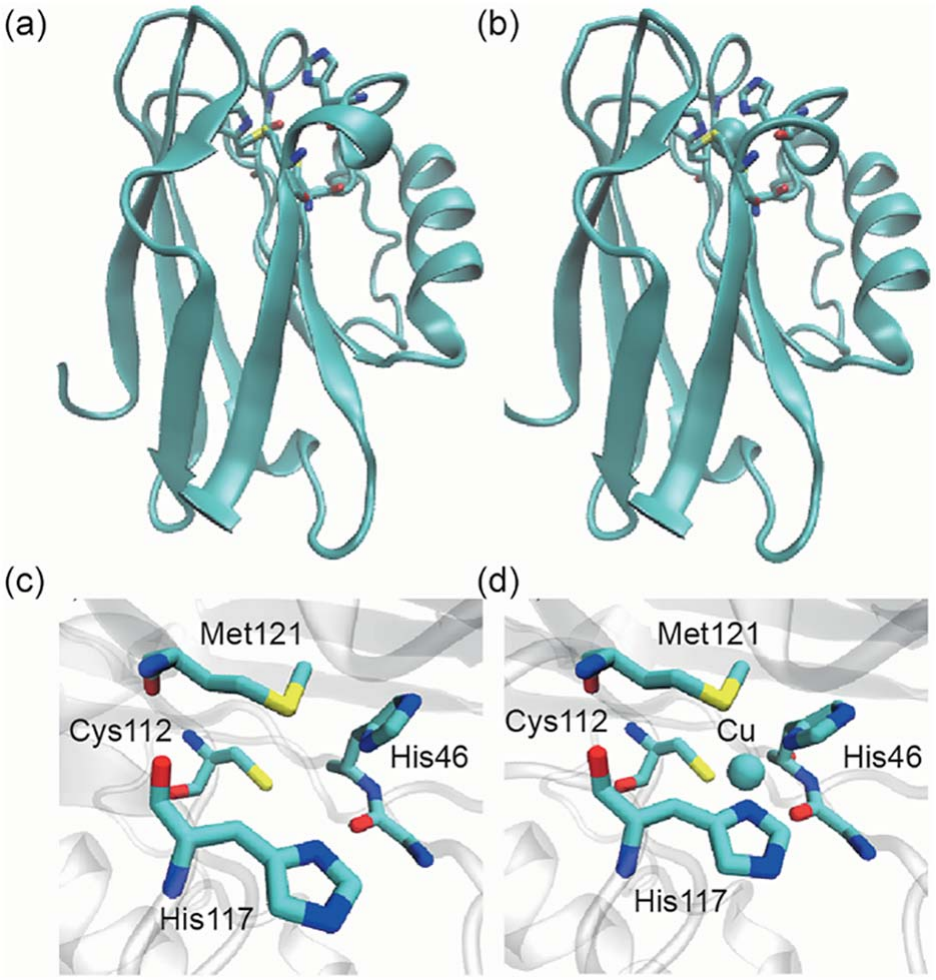
(a and b) Three-dimensional illustration showing the structure of (a) apo-azurin with a β barrel structure without Cu^2+^ and (b) azurin with Cu^2+^; the Cu^2+^-binding domain (c) without and (d) with Cu^2+^ coordination.

Typical NaYF_4_:20%Yb,2%Er UCNPs were synthesized using oleic acid as a capping ligand.^2*a*^ Upon NIR excitation (980 nm), the UCNPs exhibit multiple upconversion luminescence (UCL) bands in the green and red regions, respectively (Fig. 2). Azurin is a well-characterized, 14 kDa electron-transfer protein involved in bacterial denitrification, such as those in *Pseudomonas aeruginosa*.^12^ It has unique spectroscopic features including strong absorption (>5000 M^−1^ cm^−1^) in the UV-vis absorption spectrum and a small hyperfine splitting (∼50 × 10^−4^ cm^−1^) for EPR.^12^ In the form without Cu^2+^ binding, apo-azurin shows no absorption in the visible region. Apo-azurin could bind Cu^2+^ quite tightly through two histidines (N), a cysteine (S), and a methionine (S) (Fig. 3) and yield a significant absorbance at 625 nm with a molar extinction coefficient of 5600 M^−1^ cm^−1^,^12^ which ideally overlaps with the red emission band of the UCNPs. In this way, the red emission band at 654 nm of UCNPs is absorbed to various degrees, while its emission band at 521 nm, by contrast, is constant and serves as an internal reference for the ratiometric sensing of Cu^2+^.

**Fig. 4:**
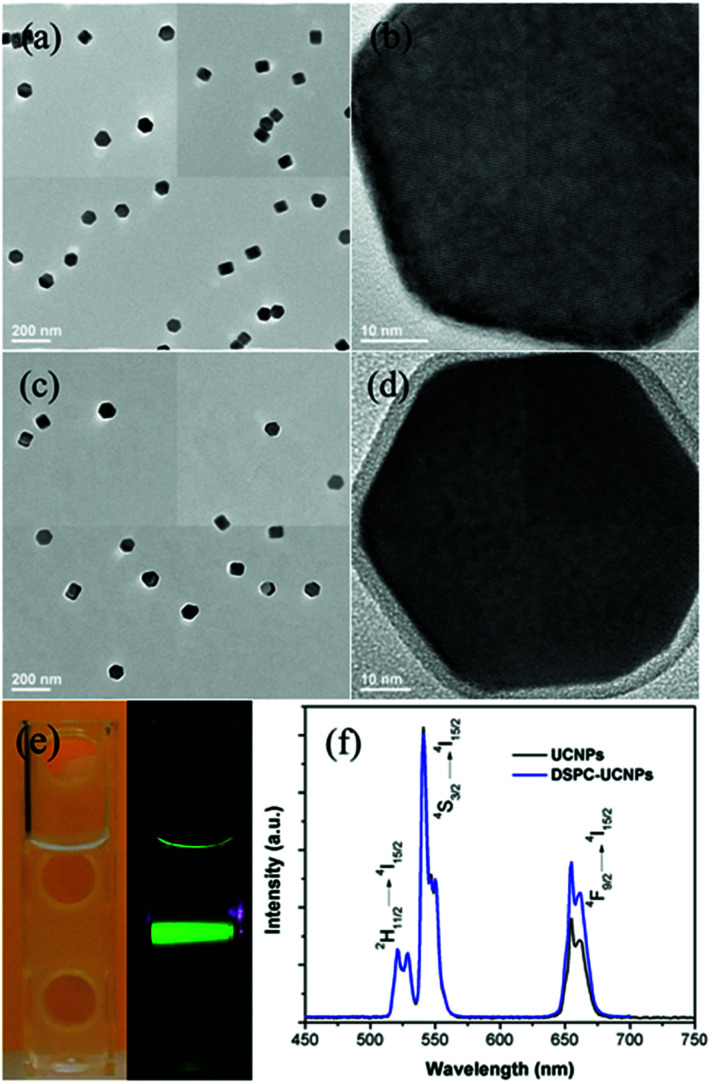
TEM images of the (a and b) as-prepared and (c and d) phospholipid DSPC coated UCNPs deposited on a TEM grid from a drop of UCNP cyclohexane solution and DSPC–UCNP water solution, respectively; (e) photographs of the water solution of DSPC–UCNPs without laser illumination and the upconverted visible luminescence under continuous-wave 980 nm laser illumination; (f) room-temperature UCL spectra of the as-prepared UCNPs in cyclohexane and DSPC–UCNPs in water under excitation at 980 nm.

The NaYF_4_:20%Yb,2%Er nanocrystals display a uniform hexagonal plate-like morphology with mean sizes of ∼60 nm (Fig. 4a and b and S1†). As they are prepared in organic solvents and capped with hydrophobic ligands, they are less water-dispersible and lack any functional groups for surface modification, which is a problem for biomedical application of this class of materials.^4*i*,4*j*^ To increase their water-dispersibility and biocompatibility, a phospholipid with a zwitterionic headgroup, distearoylphosphatidylcholine (DSPC), was used to coat UCNPs with an outer surface mimicking the functionality of the cell's external membrane, since phosphorylcholine (PC) is the major hydrophilic part of the cell outer membrane. In an aqueous medium, the fatty acid chains of the phospholipid DSPC were embedded in the hydrophobic surface of the UCNPs, and the hydrophilic phosphorylcholine groups point out toward the aqueous environment, thus forming a coating with a structure analogous to the cell membrane.^4*i*^ Representative TEM images of the resulting DSPC–UCNPs show that they remain monodisperse in water without aggregation (Fig. 4c and S1†). High-resolution TEM investigation confirms the single crystallized nanocrystals with a ∼4 nm thick uniform amorphous oleic acid/lipid layer around it (Fig. 4d). The DSPC–UCNPs could be well dispersed in water due to the phospholipids on the outer surface. Upon continuous excitation at 980 nm, the total luminescence of the DSPC–UCNPs in water appears yellow-green in color because of the combination of green and red emissions (Fig. 4e). The corresponding UCL spectrum of the DSPC–UCNPs in water is similar to that of the as-prepared UCNPs in cyclohexane, shown in Fig. 4f. Excited with 980 nm, the Yb^3+^ ions transfer energy to Er^3+^ and result in the characteristic Er^3+^ green emission bands between 514 and 534 nm and between 534 and 560 nm due to the transitions from ^2^H_11/2_ and ^4^S_3/2_ to ^4^I_15/2_, respectively, and a red emission band between 635 and 680 nm due to the transition from ^4^F_9/2_ to ^4^I_15/2_.^1*a*^ Furthermore, DSPC–UCNPs showed prominently long-term (months) stability in water and excellent resistance to aggregation due to a balanced charge of the zwitterionic surface (Fig. S2†). Considering the cell membrane mimetic surface, high water solubility and stability, and unique optical properties of DSPC–UCNPs, it will offer an ideal platform for biosensing and bioimaging.

**Fig. 5:**
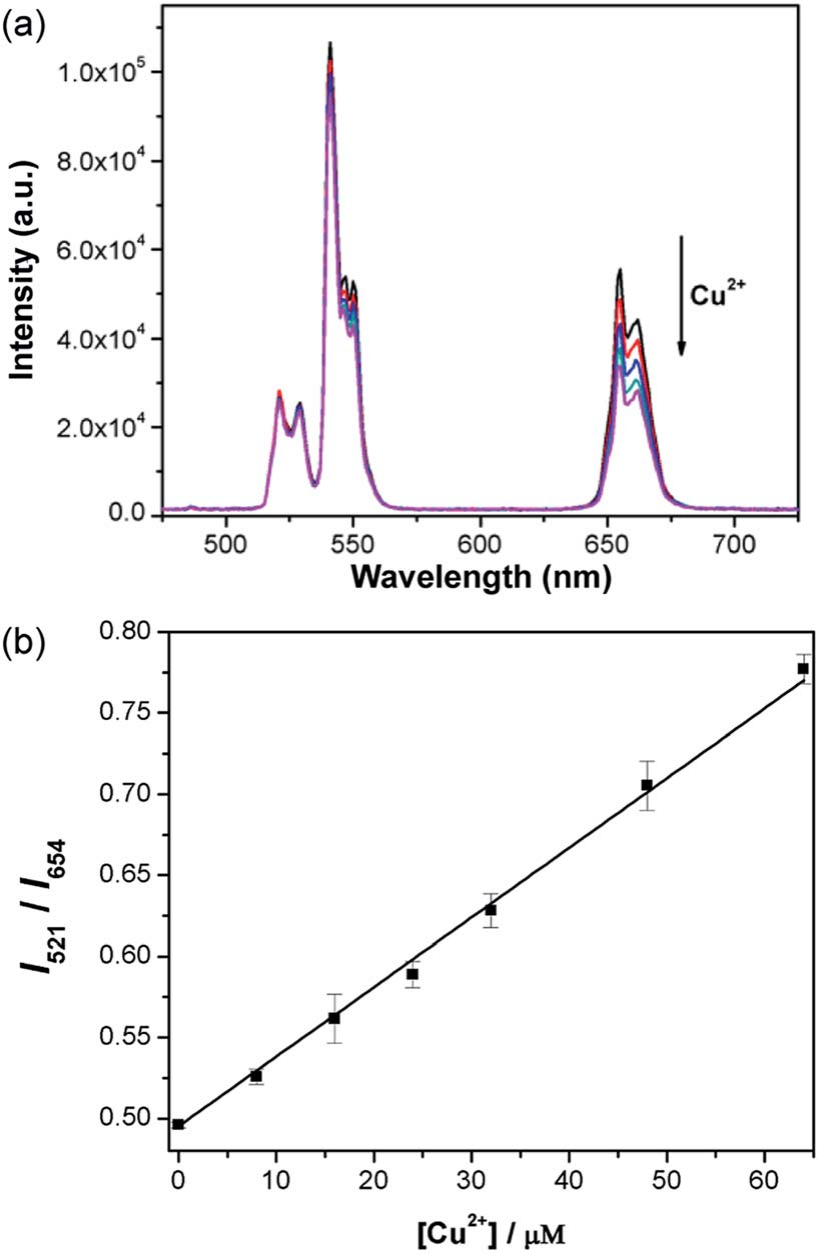
(a) UCL spectra of the apo-azurin–UCNP system (under excitation from a 980 nm laser) in the presence of varying concentrations of Cu^2+^ ions; (b) the ratio (I_521_/I_654_) of UCL intensities of the sensor system at 521 and 654 nm as a function of Cu^2+^ concentration.

Upon addition of Cu^2+^ to a solution of apo-azurin and DSPC–UCNP system, a color change from colorless to blue was observed. The intensity of the red UCL emission at 654 nm decreased gradually with increasing Cu^2+^ concentration, whereas the intensity of the green UCL band at 521 nm remained constant (Fig. 5a). Introduction of Cu^2+^ induced an increase in the spectral overlap between the red UCL emission of the UCNPs and the absorption spectrum of the metalloprotein, corresponding to decreased red UCL through the internal filter effect (IFE).^12*b*^ Since the cell membrane mimetic coating of UCNPs could prevent the adsorption of biomolecules in a biological environment, non-specific interactions were avoided between the protein and DSPC–UCNPs, making IFE play a major role. For a control experiment, the response of DSPC–UCNPs without apo-azurin on Cu^2+^ was investigated, where the Cu^2+^-dependent UCL emission change was not observed, which further confirms the importance of the protein to the sensor design (Fig. S3†). Since the green UCL emission at 520 nm is independent of Cu^2+^, this UCL emission could serve as an internal standard reference and the ratio I_521_/I_654_ was used as the detection signal. As shown in Fig. 5b, the intensity ratios of the two UCL emissions (I_521_/I_654_) gradually increase with the increasing amount of Cu^2+^. The signal ratios *versus* Cu^2+^ concentrations can be fitted to a linear regression equation with a detection limit of 2 μM, which is comparable to or lower than that of the previously reported methods.^10*d*–*f*^ In addition, the detection limit is well below the US Environmental Protection Agency and World Health Organization defined limits of 20 and 30 μM. Therefore, the detection system provides a novel way of copper detection in drinking water.

Furthermore, the time-dependent response of the system to Cu^2+^ was investigated. A response time of ∼40 seconds was observed upon addition of Cu^2+^ (Fig. S4†), which suggests that the hybrid system with a quick response can be employed as a luminescent probe for real-time detection of Cu^2+^. For the copper-bound azurin, in its oxidised (Cu^2+^) form the protein displays a strong absorption at 625 nm due to a ligand-to-metal charge-transfer transition involving mainly a sulfur 3p orbital on the Cys112 and Cu d_*x*^2^−*y*^2^_ orbital,^12^ and this absorption disappears when the Cu site is reduced into the reduced (Cu^+^) form, which suggests that the UCL intensity of UCNPs can be reversibly switched based on modulation of the protein redox states. As shown in Fig. S5,† binding with Cu^2+^ causes an obvious decrease of upconversion luminescence intensity at 654 nm while subsequent reduction of Cu^2+^ into the Cu^+^ form through the addition of a reductant (l-ascorbate acid) could bring the luminescence back to the original level. These results indicate that the azurin/UCNP sensor system shows no response to Cu^+^. This optical switch observation may provide a novel route for efficient nondestructive biomemory device fabrication.

**Fig. 6:**
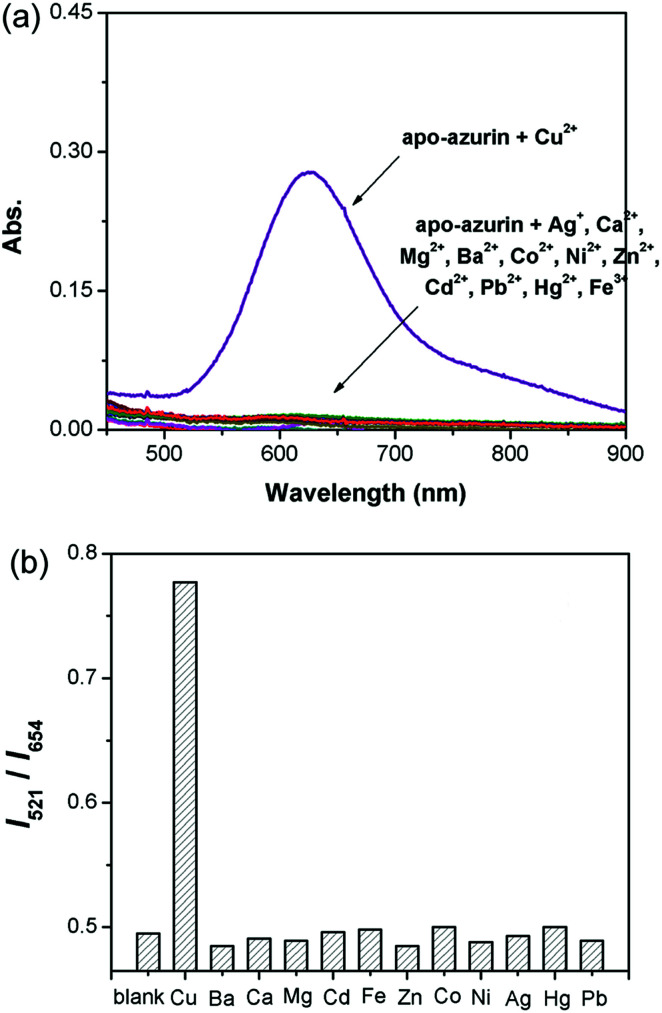
(a) Changes in the absorption spectra of apo-azurin upon addition of one eq. of various metal ions; (b) the ratio (I_521_/I_654_) of UCL intensities at 521 and 654 nm of the sensor system (under excitation from a 980 nm laser) in the presence of various representative metal ions (64 μM Cu^2+^ and 100 μM other metal ions).

High selectivity toward other biologically important cation species is necessary for a chemosensing material. The optical responses of the hybrid system were examined for other metal cations in aqueous solution. Since only Cu^2+^ could bind with apo-azurin and cause a drastic increase in the absorbance peak at *ca.* 625 nm from the formed metalloprotein (Fig. 6a), the introduction of Cu^2+^ led to significant changes in the I_521_/I_654_ ratio of UCL intensities, and no obvious UCL changes were observed for other metal ions, such as Ag^+^, Mg^2+^, Ca^2+^, Ba^2+^, Co^2+^, Ni^2+^, Fe^3+^, Pb^2+^, Zn^2+^, Cd^2+^, and Hg^2+^ (Fig. 6b). Therefore, the azurin–UCNP biosensor system described here showed highly selective ratiometric UCL sensing for Cu^2+^.

## Conclusions

In conclusion, zwitterion-coated UCNPs were synthesized through a simple biomimetic approach. By integration of the obtained water-soluble UCNPs with a redox-active copper protein, the label-free UCL sensing of Cu^2+^ with a ratiometric response was demonstrated, and it possesses attractive features including NIR excitation, absence of a luminescence background, and ease of fabrication. Owing to the facile design, the method could be readily developed to build up sensing and imaging platforms for various targets by fusion of an optical-switchable protein with UCNPs. Furthermore, since proteins have well-defined and predictable structures, and various biological activities, the protein/UCNP system offers an ideal system that should be applicable in a diverse range of areas, such as medical diagnostics, environmental monitoring, and the electronic industry. On the other hand, since zwitterionic groups on NPs will not alter the water H-bonding structure and can minimize nonspecific protein adsorption,^13^ zwitterion-coated UCNPs can be useful for various biomedical applications.

## Experimental

### Materials and reagents

All of the chemicals used were of analytical grade and were used without further purification. Rare earth chlorides, oleic acid, and 1-octadecene were purchased from Sigma-Aldrich. Phospholipid distearoylphosphatidylcholine (DSPC) was purchased from Avanti Polar Lipids. The wild-type apo-azurin was expressed and purified as previously reported.^12*a*^ β-NaYF_4_:Yb,Er was synthesized according to a previously reported method using rare earth chlorides as a precursor and oleic acid as a stabilizing agent.^14^

### Phospholipid coated UCNPs (DSPC–UCNPs)

The oleic acid capped UCNPs (*ca.* 0.05 mmol) were dissolved in 0.5 mL of chloroform and then mixed with a chloroform solution (2 mL) containing 12 mg DSPC. The solvent was evaporated slowly, and the obtained film was heated to 75 °C for a while to completely remove chloroform. The film was hydrated with water (5 mL), and the UCNPs became water-dispersible after vigorous sonication and stirring at 75 °C. The solution was centrifuged briefly and the sediment was discarded to remove large aggregates. Excess lipids were purified from DSPC–UCNPs by ultracentrifugation and washing.

### Detection

In a 1 × 1 cm quartz cuvette, a freshly prepared solution mixture (2 mL) containing 70 μM apo-azurin and 100 μL of the above nanoparticles in HEPES buffer (10 mM, pH 7.4) was used for Cu^2+^ detection, into which a small volume (2 μL) of concentrated metal stock solution was added and equilibrated for 5 min before the spectral measurements. To determine the selectivity of the sensor, 100 μM of various metal ions (M^2+^) including Ag^+^, Mg^2+^, Ca^2+^, Ba^2+^, Co^2+^, Ni^2+^, Fe^3+^, Pb^2+^, Zn^2+^, Cd^2+^, and Hg^2+^ was added to the sensor solution individually and the luminescence response was monitored.

### Instrumentation

Transmission electron microscopy (TEM) images were taken on a JEOL 2100 Cryo transmission electron microscope with an accelerating voltage of 200 kV. Fluorescence spectra were recorded on a FluoroMax-P fluorimeter (HORIBA Jobin Yvon Inc., Edison, NJ) equipped with a commercial CW IR laser (980 nm). UV/vis spectra were recorded on a Hewlett–Packard 8453 spectrometer.

## Conflicts of interest

There are no conflicts to declare.

## Supplementary Material

NA-001-C9NA00168A-s001
