# Supplementary material for: Cupredoxin engineered upconversion nanoparticles for ratiometric luminescence sensing of Cu2+
Source: Nanoscale Adv. 2019 May 13;1(7):2580–5. doi: 10.1039/c9na00168a (PMC7081933; doi:10.1039/c9na00168a)
Supplement: NA-001-C9NA00168A-s001 [file NA-001-C9NA00168A-s001.pdf]

Electronic Supplementary Material (ESI) for Nanoscale Advances.
This journal is © The Royal Society of Chemistry 2019

ELECTRONIC SUPPLEMENTARY INFORMATION FOR

Cupredoxins Engineered Upconversion Nanoparticles for Ratiometric Luminescence Sensing of Cu^{2+}

Chang Liu, YingjieYu, Daquan Chen,* Jian Zhao, Yang Yu, Lele Li* and Yi Lu*

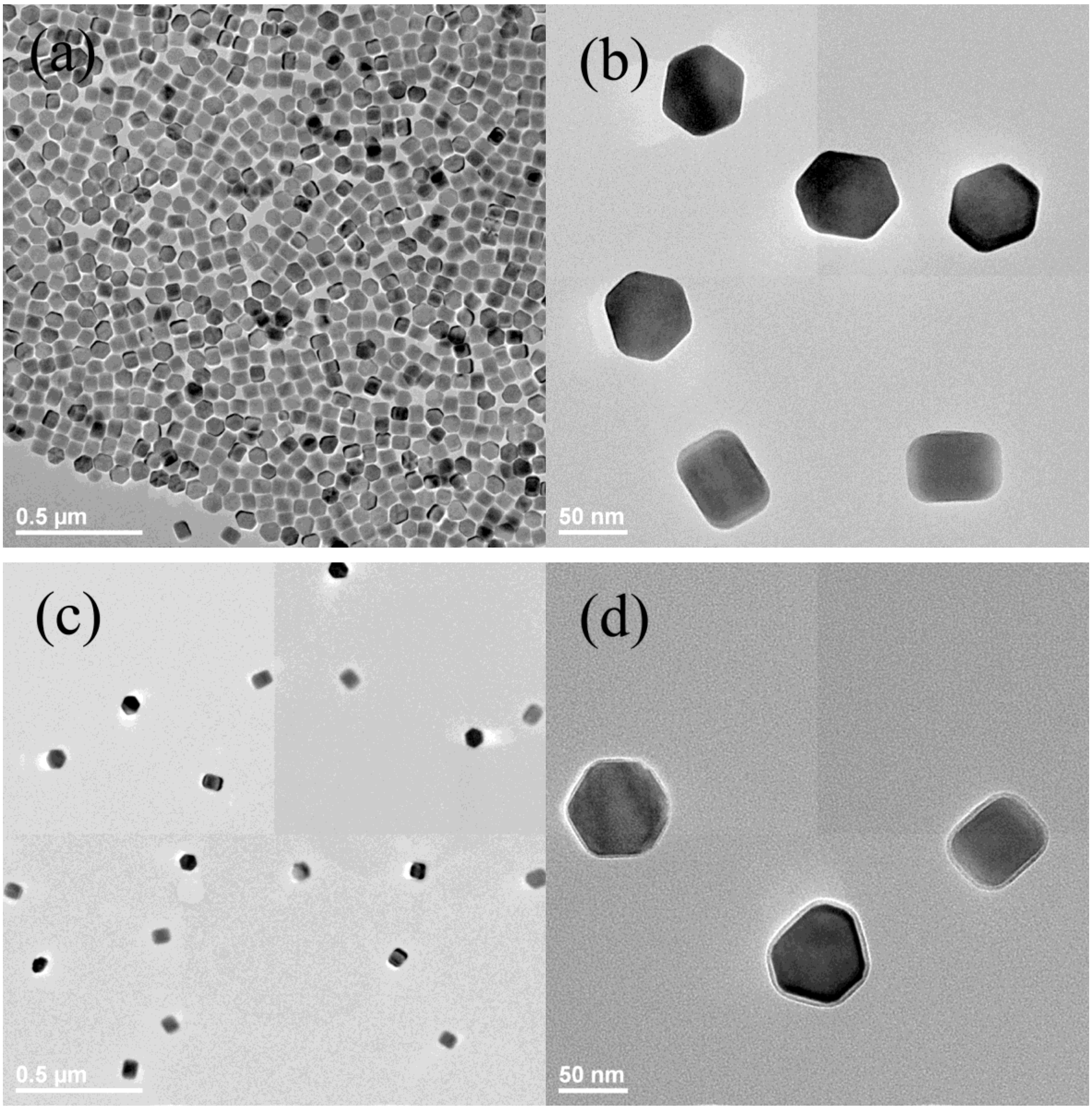

Figure S1. TEM images of the (a, b) as-prepared and (c, d) phospholipids DSPC coated UCNPs deposited on TEM grid from a drop of UCNPs cyclohexane solution and DSPC-UCNPs water solution, respectively.

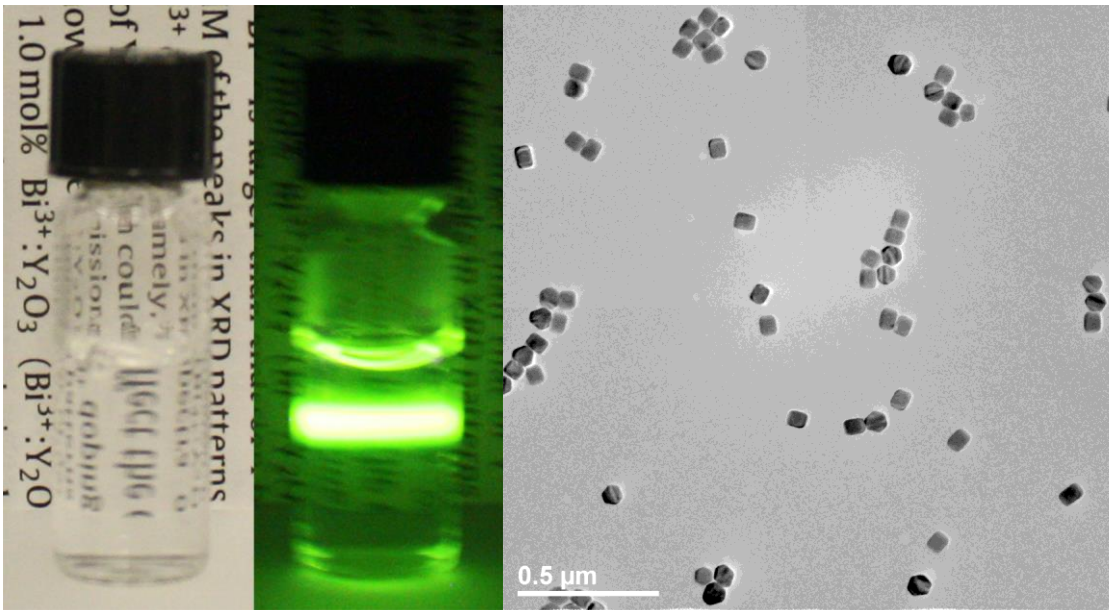

Figure S2. (Left) Photographs of the water solution of DSPC-UCNPs stored in water at room temperature for four months without laser illumination and the upconverted visible luminescence under continuous-wave 980 nm laser illumination. (Right) TEM images of the DSPC-UCNPs sample stored in water at room temperature for four months, indicating they remain monodisperse in size without aggregation.

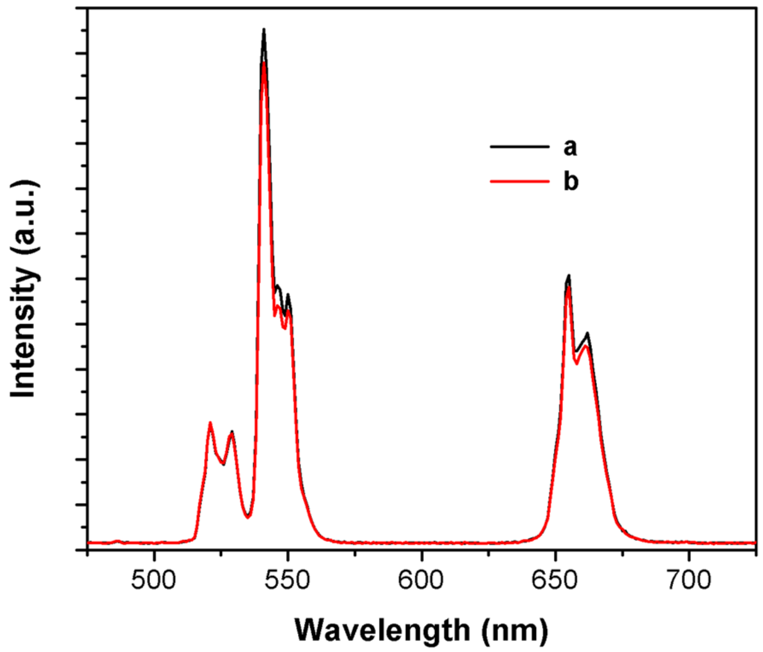

Figure S3. Upconversion luminescence spectra of the DSPC-UCNPs in aqueous solution in the absence (a) and presence (b) of 200 μM Cu^{2+}.

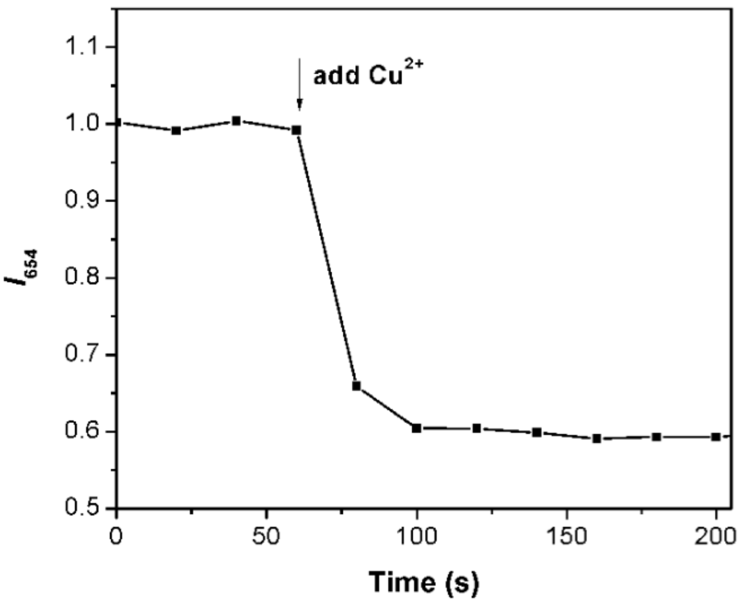

Figure S4. Time-dependent upconversion luminescence response of the sensor system with the adding of 70 μM Cu^{2+}.

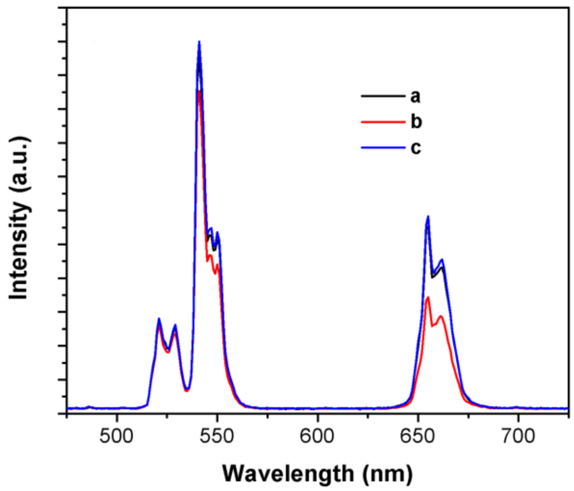

Figure S5. Upconversion luminescence spectra of the sensor system in the absence (blank line) and presence (red line) of Cu^{2+}, and Cu^{2+}-treated hybrid system upon addition of 10 equiv. L-ascorbate acid (blue line).
